# Hydrogen gas inhalation ameliorates LPS-induced BPD by inhibiting inflammation via regulating the TLR4–NFκB–IL6/NLRP3 signaling pathway in the placenta

**DOI:** 10.1186/s40001-024-01874-9

**Published:** 2024-05-14

**Authors:** Yafang Zhang, Xianhui Ren, Linli Zhang, Xinliu Sun, Wenjing Li, Yunxi Chen, Yan Tian, Zhongxia Chu, Youzhen Wei, Guo Yao, Yan Wang

**Affiliations:** 1 Department of Neonatology and NICU, The Affiliated Taian City Central Hospital of Qingdao University, Taian, Shandong China; 2 Medical Imaging Center, The Affiliated Taian City Central Hospital of Qingdao University, Taian, Shandong China; 3 Central Laboratory, The Affiliated Taian City Central Hospital of Qingdao University, Taian, Shandong China; 4 Department of Ultrasound, Taian Traditional Chinese Medicine Hospital, Taian, Shandong China; 5 Research Center for Translational Medicine, Tongji University Affiliated East Hospital, Shanghai, China

**Keywords:** Hydrogen, BPD, Placenta, Inflammation, TLR4, NFκB, NLRP3

## Abstract

**Introduction:**

Hydrogen (H_2_) is regarded as a novel therapeutic agent against several diseases owing to its inherent biosafety. Bronchopulmonary dysplasia (BPD) has been widely considered among adverse pregnancy outcomes, without effective treatment. Placenta plays a role in defense, synthesis, and immunity, which provides a new perspective for the treatment of BPD. This study aimed to investigate if H_2_ reduced the placental inflammation to protect the neonatal rat against BPD damage and potential mechanisms.

**Methods:**

We induced neonatal BPD model by injecting lipopolysaccharide (LPS, 1 µg) into the amniotic fluid at embryonic day 16.5 as LPS group. LPS + H_2_ group inhaled 42% H_2_ gas (4 h/day) until the samples were collected. We primarily analyzed the neonatal outcomes and then compared inflammatory levels from the control group (CON), LPS group and LPS + H_2_ group. HE staining was performed to evaluate inflammatory levels. RNA sequencing revealed dominant differentially expressed genes. Bioinformatics analysis (GO and KEGG) of RNA-seq was applied to mine the signaling pathways involved in protective effect of H_2_ on the development of LPS-induced BPD. We further used qRT-PCR, Western blot and ELISA methods to verify differential expression of mRNA and proteins. Moreover, we verified the correlation between the upstream signaling pathways and the downstream targets in LPS-induced BPD model.

**Results:**

Upon administration of H_2_, the inflammatory infiltration degree of the LPS-induced placenta was reduced, and infiltration significantly narrowed. Hydrogen normalized LPS-induced perturbed lung development and reduced the death ratio of the fetus and neonate. RNA-seq results revealed the importance of inflammatory response biological processes and Toll-like receptor signaling pathway in protective effect of hydrogen on BPD. The over-activated upstream signals [Toll-like receptor 4 (TLR4), nuclear factor kappa-B p65 (NF-κB p65), Caspase1 (Casp1) and NLR family pyrin domain containing 3 (NLRP3) inflammasome] in LPS placenta were attenuated by H_2_ inhalation. The downstream targets, inflammatory cytokines/chemokines [interleukin (IL)-6, IL-18, IL-1β, C–C motif chemokine ligand 2 (CCL2) and C-X-C motif chemokine ligand 1 (CXCL1)], were decreased both in mRNA and protein levels by H_2_ inhalation in LPS-induced placentas to rescue them from BPD. Correlation analysis displayed a positive association of TLR4-mediated signaling pathway both proinflammatory cytokines and chemokines in placenta.

**Conclusion:**

H_2_ inhalation ameliorates LPS-induced BPD by inhibiting excessive inflammatory cytokines and chemokines via the TLR4–NFκB–IL6/NLRP3 signaling pathway in placenta and may be a potential therapeutic strategy for BPD.

## Introduction

Owing to the lack of effective interventions to prevent preterm births and life-saving postpartum intervention, BPD has been recognized as the most frequent complication in premature infants and is accompanied by high incidence and poor prognosis [1]. It can lead to the development of chronic lung dysfunction and persistent airway and pulmonary vascular diseases, seriously affecting survival quality [2, 3].

BPD is now considered the result of an abnormal repair response to lung injury by multiple antenatal and postnatal exposures [4]. The etiology and pathogenesis of BPD are complex. (1) It is well known that inflammation is the key to the occurrence of BPD, whether prenatal or postpartum. Especially, chorioamnionitis (CAM) is expressed as the common antenatal pathway that initiates lung injury that can progress to a BPD phenotype [5]. CAM is an independent risk factor for the occurrence of BPD, as well as the most common and important factor in clinic. When the pathogen infects the amniotic cavity through ascending transmission, causing chorioamnionitis, the placenta releases a large number of inflammatory factors into the fetal blood circulation, resulting in the accumulation of inflammatory factors in the fetal lung, which lead to the decrease of the expression of lung cytokines, turn out fetal pulmonary developmental disorders. In addition, the release of placental chemokines promotes the infiltration of proinflammatory cells in fetal lung tissue, affects the development of alveoli and eventually leads to the occurrence of BPD. (2) Postpartum infection: mainly found in preterm infants with severe pneumonia, NEC, sepsis, etc. Due to immunodeficiency in preterm infants, systemic inflammatory response syndrome develops rapidly when combined with severe infection. Substantial numbers of inflammatory factors are released into the blood circulation and aggregated in the pulmonary circulation, which also leads to lack of ventilation and hypoxia to further aggravate the lung injury, resulting in the occurrence of BPD. (3) Hyperoxia: mainly occurs in long-term oxygen dependence of premature infants. Lack of relatively mature antioxidant capacity in premature infants, oxygen free radicals increase significantly when exposed to hyperoxia. Abundant oxygen free radicals accumulate in the lung, which further initiate inflammation, apoptosis and other signal pathways, resulting in lung injury and promoting the occurrence of BPD. (4) Ventilator application: due to the immature lung development of premature infants, ventilator is required to provide high airway pressure and high tidal volume to maintain normal breathing and blood oxygen saturation to rescue. Unfortunately, airway pressure damage is also accompanied by, which can induce the lung infection and oxidative stress, in turn, give rise to the obstruction of pulmonary development. (5) In addition, preeclampsia, vitamin deficiency, smoke exposure and genetic susceptibility are also high risk factors for BPD. Although the pathogenesis of BPD is complex, inflammation plays a primary role in the BPD onset. Inhibition of inflammatory responses is particularly crucial to reduce BPD.

Placenta, as a bridge between maternal to fetus and owing to the advantage of the available clinical specimens, has gradually become a hot topic for the study of perinatal neonatal diseases. With the continuous excavation of placental function, placenta not only acts as a key medium for maternal–infant nutrition exchange, but also as the regulatory hub located between the maternal and the fetus, which plays a role in the regulation of inflammation, gradually arouse widespread concern and become the focus of research. Placental components, such as proinflammatory factors and chemokines, can promote the activation of inflammatory cells such as monocytes and macrophages. On the other hand, it can enhance the migration of proinflammatory cells. Increasing evidence linking the inflammatory regulatory function in the placenta to BPD has been analyzed in a systematic review [6]. The placenta makes an adaptive response to CAM induced by LPS [7], and even placental histology is believed to predict adverse neonatal outcomes in some studies, which can cause the aggregation of placental decidual cells, amniotic cells and infiltrating macrophages and release proinflammatory cytokines and chemokines to the gestational sac [8], subsequently resulting in fetal inflammatory response syndrome (FIRS) and eventually contributing to the development of BPD [9, 10]. Concerning the vital role of the placenta in anti-inflammation, it is conceivable that alterations of the placenta can reverse the process of LPS-induced BPD and result in improving the poor prognosis that has lifelong consequences.

Hydrogen, a novel therapeutic molecule, mainly exerts anti-inflammatory, antioxidant and anti-apoptosis effects, which have been confirmed by many cellular, animal and clinical trials [11–13]. Despite many inaccuracies, inflammation scavenging ability is still the widely accepted mechanism of H_2_ [14, 15]. Lack of any known adverse effects of hydrogen makes hydrogen an ideal anti-inflammatory therapeutic modality [16, 17].

Therefore, the purpose of this study was to investigate the protective effect of hydrogen in placenta against LPS-induced BPD and to explore its underlying molecular mechanisms.

## Methods

### A neonatal rat model of BPD and experimental design

All experimental procedures were approved by the Medical Ethics Committee of the affiliated Taian City Central Hospital of Qingdao University in Shandong, China (permit numbers: 2022-07-10). Eight-week-old Sprague–Dawley (SD) rats were purchased from Shanghai JSJ. Male and female rats with a ratio of 1:2 were caged overnight, and vaginal plugs were confirmed the next morning, which was counted as E0.5. We induced CAM to generate a neonatal rat model of BPD by injecting LPS (0.2 µg/µl, 5 µl) into the amniotic fluid at E16.5. LPS from *Escherichia coli* (O55:B5) was purchased from Sigma–Aldrich (L2880, Shanghai, CHINA).

### Administration of hydrogen gas

To study the protective effect of hydrogen inhalation in the neonatal model of BPD, pregnant rats were randomly divided into three groups: a normal control group (CON group, *n* = 5), a LPS-induced BPD group (LPS group, *n* = 5), and a hydrogen administration group (LPS + H_2_ group, *n* = 5). The numbers of gestational sacs were 50 in CON group, 46 in the LPS group, and 48 in LPS + H_2_ group. After the intra-amniotic injection of LPS, pregnant rats in LPS + H_2_ group were immediately placed in a hydrogen treatment warehouse with 42% hydrogen and 21% oxygen for continuous hydrogen absorption (4 h/day) until the samples were collected. The body of the warehouse is transparent to ensure visibility.

### Animal specimen collection

The placenta and umbilical cord were collected by cesarean section at E21.5, and the weights of the neonates and placentas were recorded. The newborn rats were fed breast-feeding rats prepared in advance. The intrauterine fetal death (IUFD) rate was calculated by the number of stillbirths based on fetal counts at E16.5. The number of surviving pups was continuously counted until postnatal day (P) 14. The body weights of pups were measured at P0, P7 and P14.

The research processed the placental tissue and umbilical cord during cesarean section and collected the left lung of neonatal rats at P0, P7 and P14 for hematoxylin–eosin (HE) staining and further analysis to determine the contribution of inhaled hydrogen to BPD. Pups were euthanized at P7 and P14 by an intraperitoneal injection of 120 mg/kg (2 ml/kg) sodium pentobarbital, followed by cardiac perfusion with precooled saline. The placentas were placed in the tubes and stored frozen at − 80 °C for RNA-seq, ELISA, qRT-PCR and Western blot assays.

### Histological examinations

To intuitively evaluate the degree of LPS-induced CAM and assess the perturbed lung development, all specimens, including the placentae, umbilical cords and pup lungs, were fixed by mage-iT™ Fixative Solution (4% paraformaldehyde in PBS) (Thermo Fisher Scientific Inc, USA) according to protocol of hematoxylin–eosin (HE) Stain Kit (Solarbio Science & Technology Co., Beijing, CHINA). After washing, gradient alcohol for dehydration, transparent in xylene and subsequently paraffin-embedded and cut into 5-μm-thick sections. Dewaxing twice in xylene for 10 min, and series of alcohol (100%, 95%, 85%, 75%) for 3 min in turn to hydrate to water. Hematoxylin solution stained the nucleus for 10 min and rinsed in running tap water. Followed by differentiation and re-dyeing with Eosin solution for 30 s. Finally, samples were dehydrated, rendered transparent and sealed with neutral gum. Sections were observed under a microscope and photographed. Parameters of alveolarization: the mean linear intercept (MLI), radial alveolar count (RAC) and alveolar wall ratio (alveolar wall area/total area) were measured.

### RNA sequencing (seq)

To reveal the molecular mechanism of specific biological processes and the protective effects of inhaled H_2_ on the occurrence of LPS-induced BPD, RNA sequencing analysis of placental tissue was performed in the experiment. According to the manufacturer’s protocol, the process was as follows: the total RNA was extracted by a TRIzol reagent kit (Invitrogen, Carlsbad, CA, USA), and enriched into mRNA after the quality assessment. The enriched mRNA was then fragmented into short fragments and reverse-transcribed into cDNA using the NEBNext Ultra RNA Library Prep Kit for Illumina (NEB#7530, New England Biolabs, Ipswich, MA, USA). Subsequently, adding A base, ligating the cDNA to Illumina sequencing adapters. The target fragment was recovered by agarose gel electrophoresis and amplified by PCR, ultimately completing the preparation of the whole library, and the constructed library was sequenced using an Illumina Novaseq6000 by Gene Denovo Biotechnology Co. (Guangzhou, China).

Furthermore, performing the biological analysis process to identify metabolic signaling pathways involved. A FPKM (fragment per kilobase of transcript per million mapped reads) value was calculated to quantify the expression abundance and variations by RSEM software. To identify the differentially expressed genes, we performed the analysis by DESeq2 software between two different groups. Parameters of the genes/transcripts were considered differentially statistically significant when *q*-value < 0.05 (the corrected *p*-values obtained by the BH algorithm) and absolute fold change ≥ 2. Differentially expressed genes were mapped to GO terms in the Gene Ontology database (http://www.geneontology.org/), gene numbers were calculated for every term, and significantly enriched GO terms in DEGs compared to the genome background were defined by a hypergeometric test. Kyoto Encyclopedia of Genes and Genomes (KEGG) pathway enrichment analysis was performed to identify significantly enriched metabolic pathways and signal transduction pathways in DEGs.

### Quantitative real-time polymerase chain reaction (qRT-PCR) assay

The mRNA levels of dominant differentially expressed genes screened by RNA-seq were verified by qRT-PCR. Total RNA was extracted from 100 mg of placental tissue using the TRIzol method (Invitrogen, Life technologies, USA), and was reverse-transcribed to cDNA using HiFiScript gDNA Removal RT MasterMix (Cwbio Bio Inc., Beijing, CHINA). The qRT-PCR assay was performed with Roche LightCycler 480 II Real-Time PCR System with MagicSYBR Mixture (Cwbio Bio Inc., Beijing, CHINA). Each sample was configured to a 25-ul reaction volume with β-actin as a reference gene. Reaction conditions were as follows: pre-denaturation at 95 °C for 10 min, followed by 45 cycles of denaturation at 95 °C for 5 s, annealing at 60 °C for 30 s. The melt curve was set with the parameters (95 °C for 15 s, 60 °C for 1 min, 95 °C for 15 s and 50 °C for 30 s). The design of experimental primers followed the basic principles of primer design: the primer length was 18–25 bp, and the gap between sense and anti-sense was less than 3 bp. The length of the amplified product should be controlled within 300 bp. Four bases should be randomly distributed to avoid base accumulation. Selecting the G and C bases at the 3′ end of the primer avoid autohybridization binding and dimerization. Melting temperature (Tm): sense and anti-sense should have similar Tm, with a difference of less than 5 °C. Research protocol: in the ‘Nucleotide’ section of NCBI, input the gene and species names, then download the nucleotide sequences, and use the BLAST tool on NCBI for sequence alignment. After obtaining the gene sequences, Primer Premier 5 software was used to design primers by analyzing dimers, false promoters, cross dimers, and hairpins to analyze their specificity. The sequence of primers, product length and optimal annealing temperature (Ta opt) are shown in Table 1.

**Table 1:** The sequence, product length and Ta opt of primers used for qRT-PCR analysis

Gene	Primer sequence 5′ to 3′	Product length	Ta Opt
TLR4	Sense 5′-TGGTGGCTGTGGAGACAAAAATGAC-3′	281	53.1
	Anti-sense 5′-CTGAAAGGCTTGGGCTTGAATGGAG-3′		
Nfκbiα/IκBα	Sense 5′-CTGTACGCCCCAGCATCT-3′	123	53.2
	Anti-sense 5′-GCACCCAAAGTCACCAAG-3′		
NF-κBp65/Rela	Sense 5′-ATCCCTGCTTCCCCTTTCTC-3′	88	52.5
	Anti-sense 5′-CTGTCTTATGGCTGAGGTCTGGT-3′		
IL-6	Sense 5′-ATTGTATGAACAGCGATGATGCAC-3′	150	51.3
	Anti-sense 5′-CCAGGTAGAAACGGAACTCCAGA-3′		
CXCL1	Sense 5′-ACTCCAGCCACACTCCAACA-3′	190	57.3
	Anti-sense 5′-GCGGCATCACCTTCAAACTC-3′		
CCL2	Sense 5′-GTGCTGACCCCAATAAGGAA-3′	116	49.9
	Anti-sense 5′-TGCTGAAGTCCTTAGGGTTGA-3′		
NLRP3	Sense 5′-TGAAGAGTGTGATCTGCGGAAAC-3′	124	53.5
	Anti-sense 5′-GAAAGTCATGTGGCTGAAGCTGT-3′		
Casp1	Sense 5′-CGGGCAAGCCAGATGTTTAT-3′	193	52.5
	Anti-sense 5′-AACCACTCGGTCCAGGAAATG-3′		
IL-18	Sense 5′-GCAGTAATACGGAGCATAAA-3′	177	50.3
	Anti-sense 5′-ATCCTTCACAGATAGGGTCA-3′		
IL-1β	Sense 5′-TTCTTTGAGGCTGACGGACC-3′	255	54.7
	Anti-sense 5′-TGAGTCACAGAGGATGGGCT-3′		
β-actin	Sense 5′-AGGGAAATCGTGCGTGACAT-3′	150	54.6
	Anti-sense 5′-GAACCGCTCATTGCCGATAG-3′		

### Western blot

Western blot analysis was performed on placental tissues from CON, LPS, LPS + H_2_ groups. The placental tissue was rinsed in precooled saline to remove blood and placed in an EP tube with a steel bead. 500ul lysate including phosphatase inhibitor cocktail, protease inhibitor cocktail and RIPA lysis buffer with 1:100 ratio were added to the EP tube to homogenize for 50 s at 40 HZ, then incubated for 30 min (− 20 °C for 10 min and 4 °C for 20 min). The protein supernatant was extracted by centrifugation at 12,000 rpm 4 °C for 30 min. Adopting the BCA Protein Assay Kit (Thermo Fisher Scientific, MA, United States) to quantify the protein concentration and diluted by using the Omni-Easy™ Protein Sample Loading Buffer to guarantee all samples a final protein concentration of 2 ug/ul based on the calculated protein concentration. The extracted total protein was denatured by boiling for 5 min at 100 °C after addition of protein 4× Buffer. The target proteins were isolated from 40 μg (loading amount was 40ug with volume of 20 ul) of protein samples using the Bio-Rad system (Bio-Rad, Shanghai, USA) with 10% PAGE Gel Fast Preparation Kit (Epizyme Biotech., Shanghai, CHINA) (the parameters of electrophoresis: voltage 80 V run to the end of the concentrated gel, and adjust to 160 V until the bottom of the glass plate in Tris/Glycine/SDS Running Buffer), and next, transferred to PVDF membranes using precooled transferring buffer (The transfer parameter was set as current 300 mA and transfer times were set according to the molecular weight of primary antibody with 1:1 ratio) and blocked in 5% skimmed milk for 3 h at least. The PVDF membranes were incubated overnight with different antibodies at 4 °C: Rabbit monoclonal to NLRP3 (Abcam, ab263899, USA, 1:1000), Rabbit polyclonal to TLR4 (Abcam, ab217274, USA, 1:1000), Rabbit monoclonal to NF-kB p65 (phospho S536) (Abcam, ab76302, USA, 1:1000), Rabbit Anti-NFκB p65 antibody (Bioss, bs-0465R, CHINA, 1:800), Anti-CASP1 Rabbit polyclonal antibody (Merck, SAB5701133, CHINA, 1:1000), IκBα Rabbit pAb (ABclonal, A1187, CHINA, 1:1000), Phospho-IκBα-S32 Rabbit mAb (ABclonal, AP0707, CHINA, 1:1000) and β-actin Rabbit Monoclonal Antibody (Beyotime, AF5003, CHINA, 1:2000) and incubated with HRP-labeled Goat Anti-Rabbit IgG (H+L) (Beyotime, A0208, CHINA, 1:1000) for 2.5 h at least at room temperature. β-actin was selected as the internal reference protein, which is not only a protein encoded by the housekeeper gene, but also highly conserved and expressed relatively stable. The expression of β-actin was not affected by hydrogen administration and inflammation. In addition, the molecular weight of β-actin was 42 kDa, suitable to distinguish the target protein.

Using the Omni-ECL™Enhanced Pico Light Chemiluminescence Kit (Epizyme Biotech., Shanghai, CHINA) indicated the positive target strips. The relative protein expression used ALPHA Imager HP Gel imaging system and Image J software.

### Cytokine and chemokine detection

To validate the levels of inflammatory response in the placenta, we used ELISA to measure proinflammatory cytokines and chemokines in placenta. Placental tissue was accurately weighed, rushed with balls at 4 °C, and stored overnight at − 20 °C. After two freeze–thaw cycles, the supernatant was removed after the homogenates were centrifuged at 5000×*g* for 5 min at 4 °C. The samples to be tested were separated and stored at − 20 °C. The protein concentration was determined by a BCA protein assay kit (Thermo Fisher Scientific, MA, United States). Experimental protocols of different inflammatory factors were subject to the ELISA kit instructions. The kit had been coated in the reaction well and processed with antibodies. The steps were as followed: add sample: blank well, fold ratio diluted standard well, add 100 μl of appropriately diluted sample to the reaction well and incubated at 37 °C for 1.5 h. Then washed to react to the diluted biotinylated antibody working solution at 37 °C for 1 h. Followed by reacting 30 min with 100 μl of the working solution of the enzyme conjugate after washing. 100 μl of TMB substrate solution was added to chromogenic reaction for 10–30 min, based on the obvious color gradient which appeared in the diluted standard well to terminate by adding 100 μl of 2 M sulfuric acid. The OD value of each reaction well was measured on the enzyme meter, the standard curve was drawn, and then the sample concentration was calculated according to the standard curve equation. The contents of IL-6, CCL2, CXCL1, IL-1β and IL-18 in placental tissues were determined by ELISA kits, including IL-6 uncoated ELISA Kit (Cat. no. 88-50625, Thermo Fisher Scientific, MA, United States), CCL2 ELISA Kit (Cat. no. EK387-96, MULTI Scientific, Hangzhou, China), CXCL1 ELISA Kit (Cat. no. EK396, MULTI Scientific, Hangzhou, China), IL-1β ELISA Kit (Cat. SEA563Ra, USCN KIT INC. Wuhan, China) and IL-18 ELISA Kit (Cat. SEA064Ra, USCN KIT INC. Wuhan, China).

### Statistical analysis

Statistical analysis was performed with the GraphPad Prism 8.0 database. All results are presented as mean ± SD, and *p* < 0.05 was considered significant. All data were analyzed using one-way analysis of variance (ANOVA) with Dunnett, except that GO and KEGG enrichment analyses in RNA-seq were performed using hypergeometric test and BH algorithm. Pearson correlation analysis was used to measure the relationship between NBW and placental weight and the relationship between the levels of TLR4–NFκB–IL6/NLRP3 proteins and inflammatory factors.

## Results

### H_2_ attenuated the abnormal intrauterine development induced by LPS

The neonatal outcome results were checked immediately after birth and are displayed in Table 2. Among the maternal baseline characteristics, the intrauterine fetal death (IUFD) rates were 3/50 (6%) in the CON group, 18/46 (39%) in the LPS group, and 13/48 (27%) in the LPS + H_2_ group. Moreover, there was no significant difference in maternal weight or the number of amniotic sacs.

**Table 2:** Maternal baseline characteristics in different groups

	CON	LPS	LPS + H_2_
Maternal rat (*n*)	5	5	5
Maternal weight (g)	298.80 ± 7.83	301.00 ± 8.25	285.60 ± 5.44
Gestational sacs (*n*)	50	46	48
Fetal death (*n*)	3	18	13
IUFD rate (%)	6	39	27

### H_2_ inhalation improved the poor outcomes of neonatal rats

**Fig. 1:**
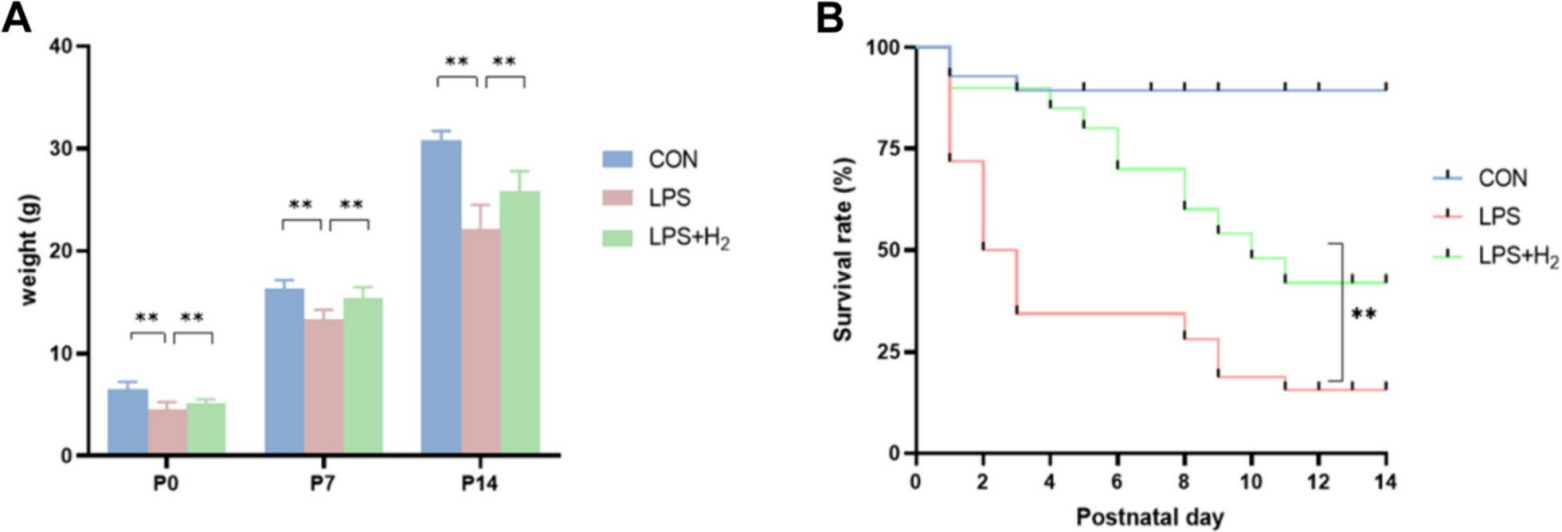
The outcomes of neonatal rats dynamically from P0 to P14. **A** Body weights of pups at P0 (*n* = 15 for CON, 15 for LPS, and 15 for LPS + H_2_) and P7 (*n* = 15 for CON, 12 for LPS, and 15 for LPS + H_2_) and P14 (*n* = 12 for CON, 9 for LPS, and 12 for LPS + H_2_). Data are presented as the mean ± SD using ANOVA followed by Tukey’s multiple comparison test. *p < 0.05, **p < 0.01 vs LPS group. **B** Kaplan–Meier survival curves of pups from P0 to P14 (*n* = 47 for CON, 28 for LPS and 35 for LPS + H_2_). A significant difference was observed by the log-rank test (*p* = 0.0017)

Similarly, the pups in the LPS group represented a growth-restricted state in the postnatal days in Fig. 1, mainly in the lower body weight at P0–P14 (Fig. 1A) and lower survival rate up to P14 (Fig. 1B). However, dysplasia and the lower survival ratio were all improved by hydrogen in the LPS + H_2_ group (*p* < 0.01).

### Positive correlation between placental weight and BW

**Fig. 2:**
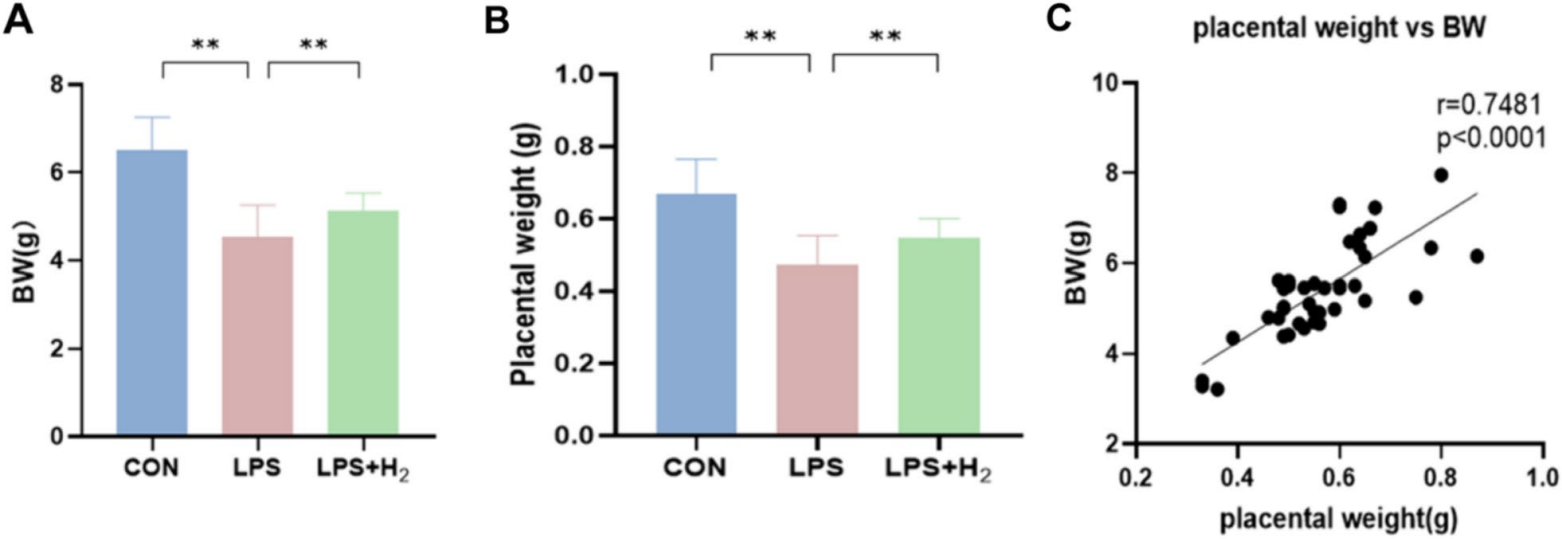
The neonatal baseline characteristics in the CON, LPS and LPS + H_2_ groups. **A**, **B** Birth weight (BW) and placental weight of pups (a total of 15 newborn rats were selected from each group (3 newborns were randomly selected from each maternal rat); *n* = 15 for CON, 15 for LPS and 15 for LPS + H_2_). *p < 0.05, **p < 0.01 vs LPS group. **C** Relationship between BW and placental weight in all groups (*n* = 45), *r* = Pearson’s correlation coefficient

Compared with that in the CON group, the BW in the LPS group was significantly lower, as shown in Fig. 2A. Similarly, the average placental weight in the LPS group was also at a lower level, as shown in Fig. 2B. Interestingly, the above abnormalities were improved after hydrogen inhalation (*p* < 0.05). A positive relationship between placental weight and BW was found in pups at P0 (*r* = 0.7528; *p* < 0.0001; Fig. 2C).

### H_2_ inhalation significantly relieved LPS-induced perturbed lung development

**Fig. 3:**
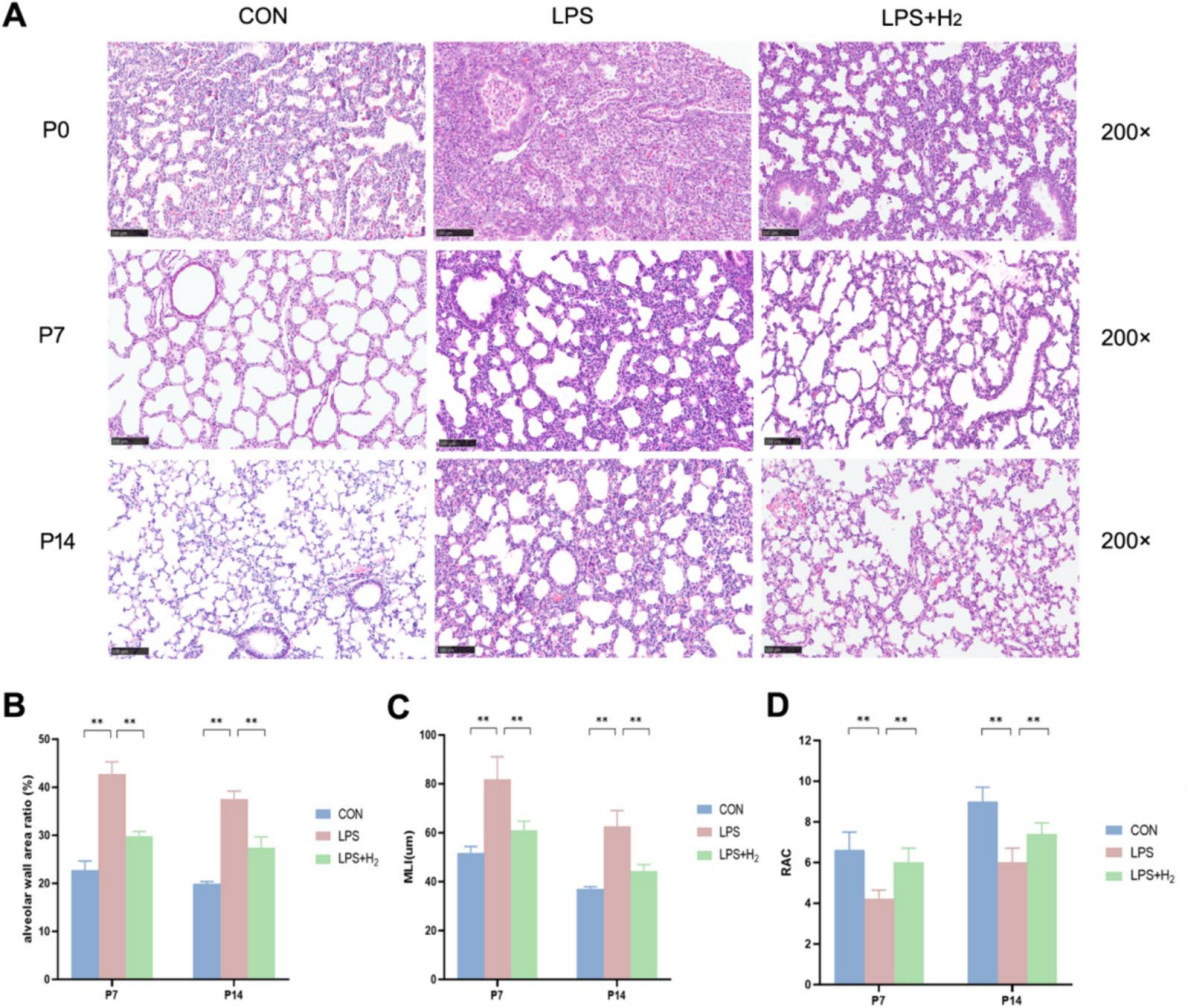
Lung pathological histology in the CON, LPS and LPS + H_2_ groups. **A** HE staining of lung tissues at P0, P7 and P14 (scale bar, 100 µm). **B** The alveolar wall area ratio: Image J software was used to quantitatively calculate the alveolar wall area and total area, and then the ratio was calculated to analyze the significant difference in the three groups (*n* = 5). **C**, **D** Graphic representation of the abundance of the average mean linear intercept (MLI) and radial alveolar count (RAC) at P7 and P14 in the three groups (*n* = 5). *p < 0.05, **p < 0.01 vs LPS group

Perturbed lung development with an obvious lag was demonstrated in the LPS group by histological examination. LPS treatment abnormally increased the alveolar size, resulting in a heterogeneous alveolar and severe inflammatory response with a large amount of polymorphonuclear leukocyte infiltration. Fortunately, hydrogen normalized the alveolar sizes and pulmonary septum and evidently reduced inflammatory cell infiltration (Fig. 3A). Quantitatively, the parameters of alveolarization, the alveolar wall area ratio and the MLI, were increased by LPS, and the RAC was decreased by LPS. In contrast, the above disorder induced by LPS was normalized by hydrogen (Fig. 3B–D).

### Hydrogen inhalation inhibited the excessive inflammatory response of LPS-induced placenta and fetus

**Fig. 4:**
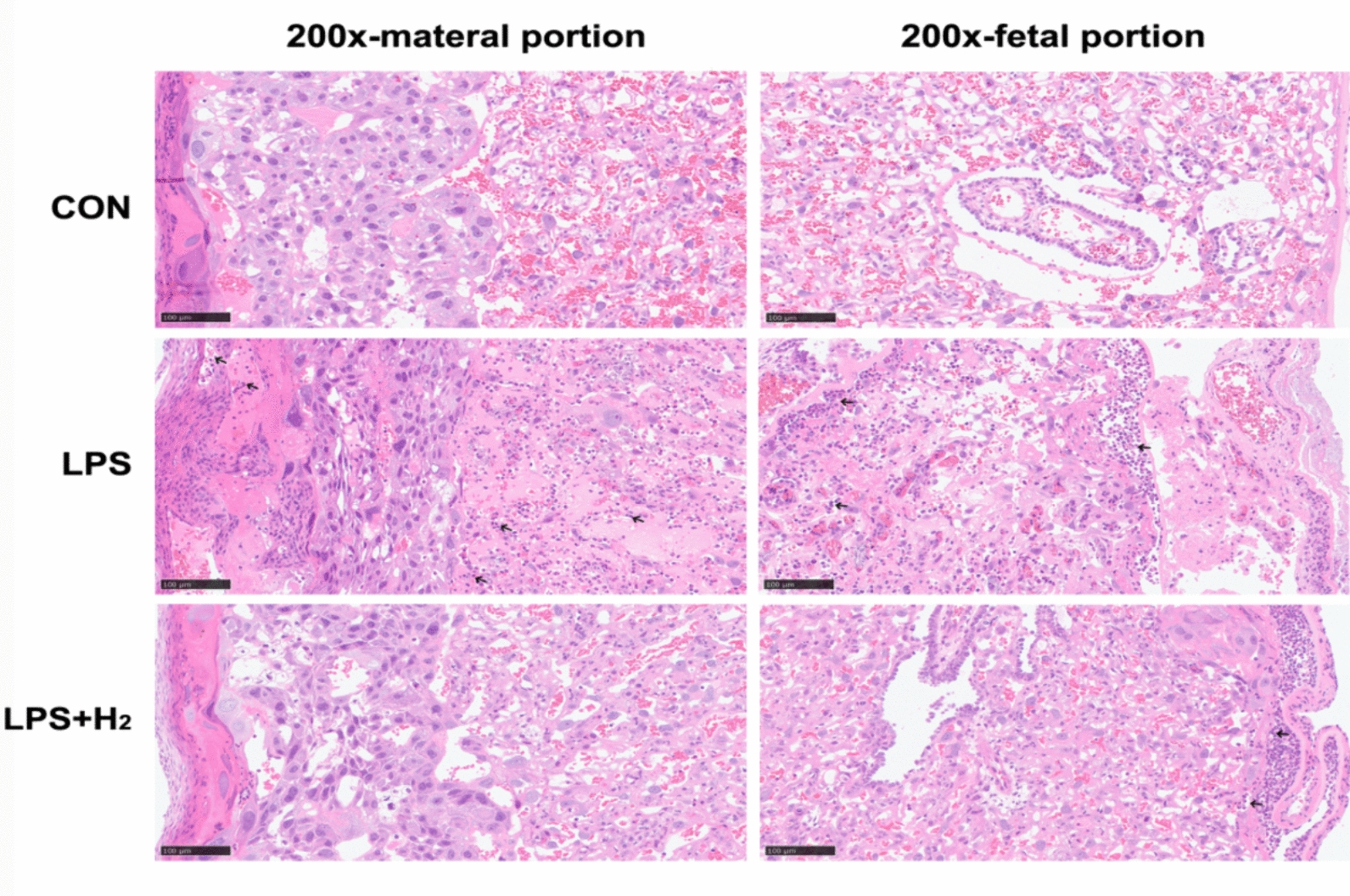
Placental histologies by HE staining in the CON, LPS and LPS + H_2_ groups. The 200× images show the maternal portion and fetal portion to present the degree of polymorphonuclear leukocyte infiltration. Black arrows indicated polymorphonuclear leukocyte infiltration in different portion of placenta

**Fig. 5:**
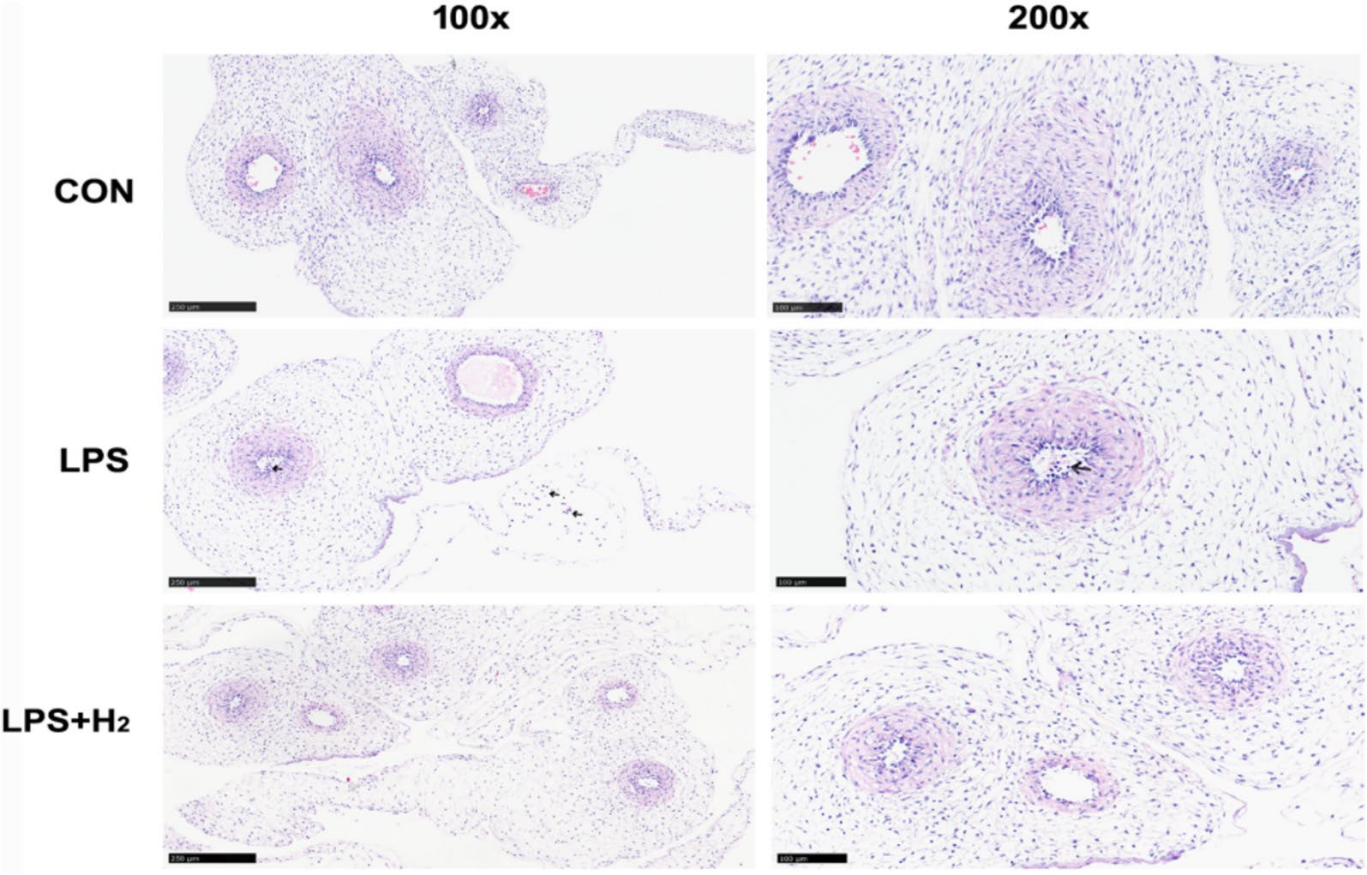
Umbilical cord histologies by HE staining in the CON, LPS and LPS + H_2_ groups. Inflammatory cells were pointed out with black arrows

Placentae in the LPS group displayed severe CAM, which manifested as a large amount of polymorphonuclear leukocyte infiltration (200×, Fig. 4), involving the lower pole of the amniotic sac, the intervillous space, the chorionic plate, decidua and even the whole layer of the placenta. Interestingly, upon administration of H_2_, the infiltration degree was remarkably reduced, and the infiltration range significantly narrowed; only the amniotic sac and chorionic plate were involved. Similarly, this phenomenon was also presented in the HE staining of umbilical cord, which reflected the degree of inflammation in the fetus. Unlike the CON group, inflammatory cells were scattered in the umbilical artery (200×, Fig. 5) and surrounding amniotic membrane (100×, Fig. 5) in the LPS group but not in the LPS + H_2_ group.

### The TLR4–NFκB–IL6/NLRP3 signaling pathway was involved in hydrogen inhalation rescue from LPS-induced inflammatory imbalance in the placenta

**Fig. 6:**
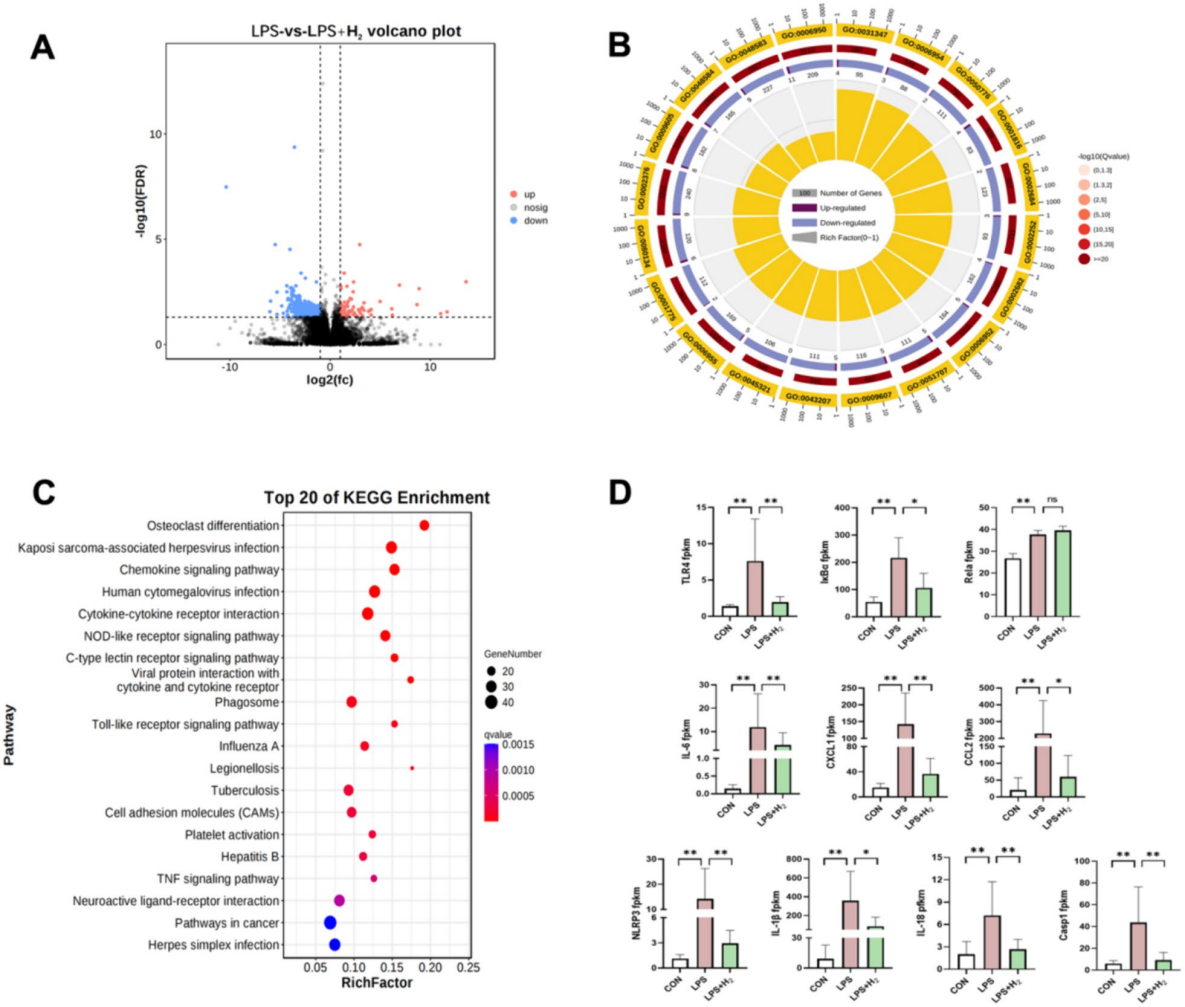
Hydrogen reversed the dysregulated placental genes of the TLR4–NFκB–IL6/NLRP3 inflammatory signaling pathway in response to LPS. **A** Placentae in the LPS group and LPS + H_2_ group were sent for RNA-seq. A volcano plot map of the differentially expressed genes was identified with cut-off values of *q*-value < 0.05 and |log(fc)|> 1, in which red represented upregulated genes and blue represented downregulated genes. **B**, **C** GO and KEGG enrichment analyses were performed with the differentially expressed genes. **D** The FPKM levels of the TLR4–NFκB–IL6/NLRP3 signaling pathway (*n* = 4). *p < 0.05, **p < 0.01 vs LPS group, *q*-value: the corrected *p*-values obtained by the BH algorithm were conceptually equivalent to adjusted *p*-value

To further verify the underlying mechanisms of the H_2_ effect, the transcriptome in the CON, LPS and LPS + H_2_ groups was determined via RNA-seq. The RNA-seq data have been deposited in the NCBI Sequence Read Archive (SRA) database, with accession number PRJNA901070. Differentially expressed genes indicated that H_2_ could have a therapeutic effect on LPS-induced BPD via various molecular mechanisms. A total of 671 genes with significant differences were screened out when comparing the LPS + H_2_ group with the LPS group, including 78 upregulated and 593 downregulated genes (Fig. 6A). Significantly enriched GO terms circular bar plot identified the outstanding ‘inflammatory response (GO:0006954)’ biological processes in placenta (Fig. 6B). To further elucidate the mechanism, KEGG pathway analysis was also applied to analyze the dysregulated genes involved in various signal transduction pathways. Among the top 20 KEGG enrichments, the Toll-like receptor signaling pathway was strikingly located (Fig. 6C).

Meanwhile, we screened multiple differentially expressed genes based on TLR-mediated inflammatory signaling pathway, and presented them as the FPKM levels (Fig. 6D). The increased mRNA levels of TLR4, NF-κB inhibitor α (IκBα), NLRP3 and Casp1 in LPS placenta were attenuated by H_2_ inhalation. The downstream targets, overactive inflammatory cytokines/chemokines (IL-6, IL-18, IL-1β, CCL2 and CXCL1) were also decreased in LPS + H_2_ group.

### H_2_ inhalation inhibited inflammatory response via TLR4–NFκB–IL6/NLRP3 inflammatory signaling pathway in placenta

#### Alterations of mRNA levels of TLR4–NFκB–IL6/NLRP3 inflammatory signaling pathway in placenta

The RNA-seq results in this research revealed that the TLR4–NFκB–IL6/NLRP3 signaling pathway was involved in the inflammatory response. Therefore, it was hypothesized that H_2_ may function via the TLR4–NFκB–IL6/NLRP3 inflammatory signaling pathway. So, the study expanded the number of samples, and validated the mRNA expression by qRT-PCR analysis.

**Fig. 7:**
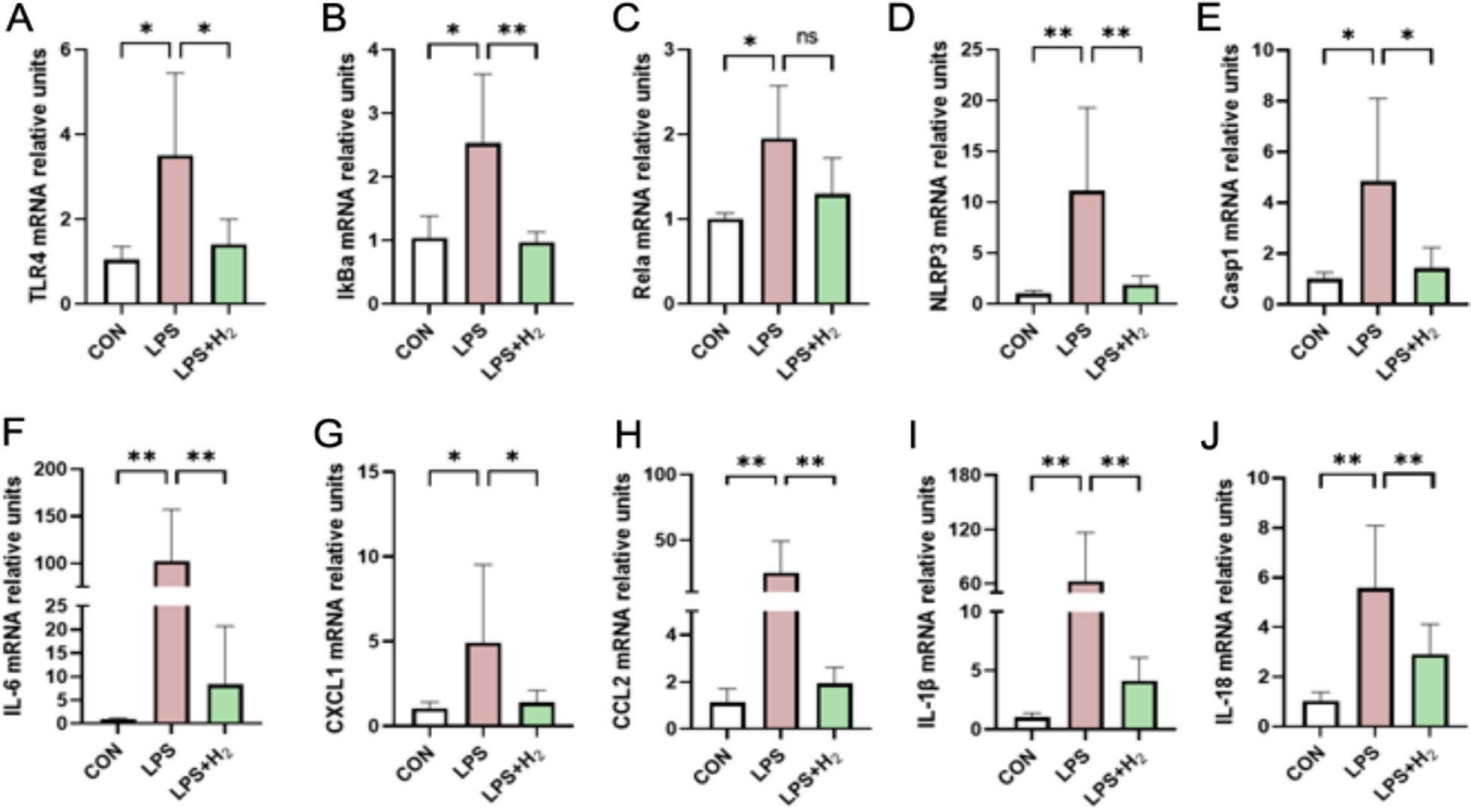
mRNA levels of TLR4–NFκB–IL6/NLRP3 inflammatory signaling pathway in placenta. qRT-PCR analysis of relative mRNA levels of the genes in placenta. Bar graph shows mean ± SD of eight samples from each group, respectively. Data were displayed as mean ± SD. *p < 0.05, **p < 0.01 vs LPS group

As shown in Fig. 7, placenta in LPS group had significantly higher mRNA levels of TLR4, IκBα, NF-κB p65(Rela), NLRP3, Casp1, IL-6, CXCL1, CCL2, IL-18 and IL-1β (*p* < 0.05; Fig. 7A–J) compared to CON group. While, which were inhibited after inhaled hydrogen except for Rela (*p* < 0.05; Fig. 7A–J).

### H_2_ inhalation moderated the increased protein levels of the TLR4–NFκB–IL6/NLRP3 signaling pathway and scavenged excessive placental inflammatory cytokines and chemokines

**Fig. 8:**
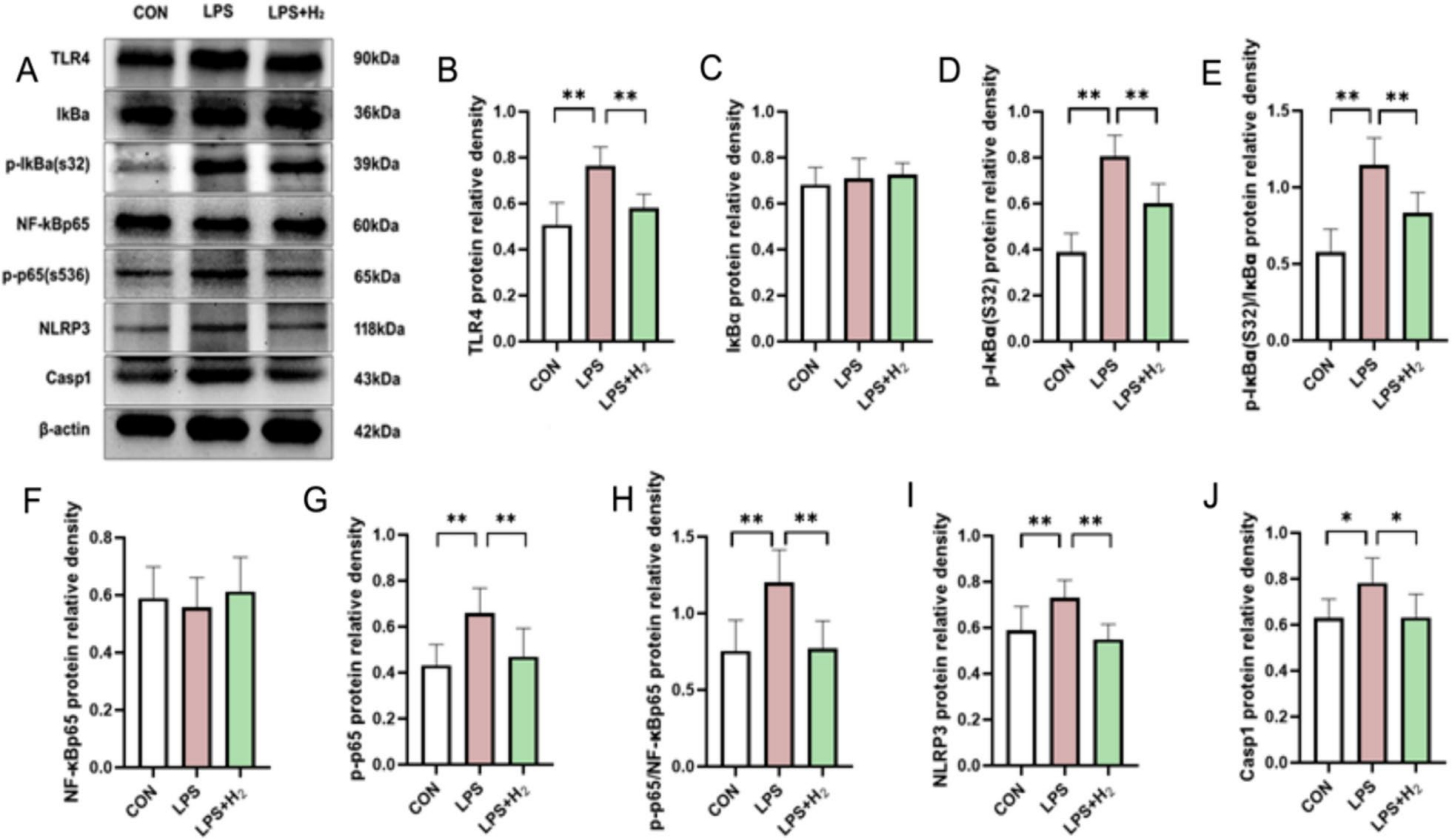
Hydrogen downregulated the placental TLR4–NFκB-IL6/NLRP3 inflammatory signaling pathway to rescue from LPS-induced BPD. **A** A representative image of Western blots for TLR4, IκBα, Phospho-IκBα (Ser32) ((p-IκBα(s32)), NF-κB p65, NF-kB p65 (phospho S536)(p-p65), NLRP3 and Casp1 in placenta from each group. β-actin was used to verify equivalent loading. Graphic representation of relative density of TLR4 (**B**), IκBα (**C**), p-IκBα(s32) (**D**), NF-κB p65 (**F**), p-p65(s536) (**G**), NLRP3 (**I**) and Casp1 (**J**) normalized to β-actin. Graphic representation of relative abundance of p-IκBα(s32)/IκBα (**E**) and *p*-p65/NF-κB p65 (**H**). Data were presented as mean ± SD of 8 samples from each group. *p < 0.05, **p < 0.01 vs LPS group

Compared to the CON group, the placental protein level of TLR4, p-IκBα(s32), p-IκBα(s32)/IκBα, p-p65(s536), p-p65/NF-κBp65, NLRP3 and Casp1 in the LPS group was remarkably increased in response to LPS (*p* < 0.05; Fig. 8). Fortunately, inhaled H_2_ moderated the above disorder (*p* < 0.05; Fig. 8). In addition, there was no significant difference in total NF-κBp65 and IκBα protein levels between the three groups (*p* > 0.05; Fig. 8C and F).

**Fig. 9:**
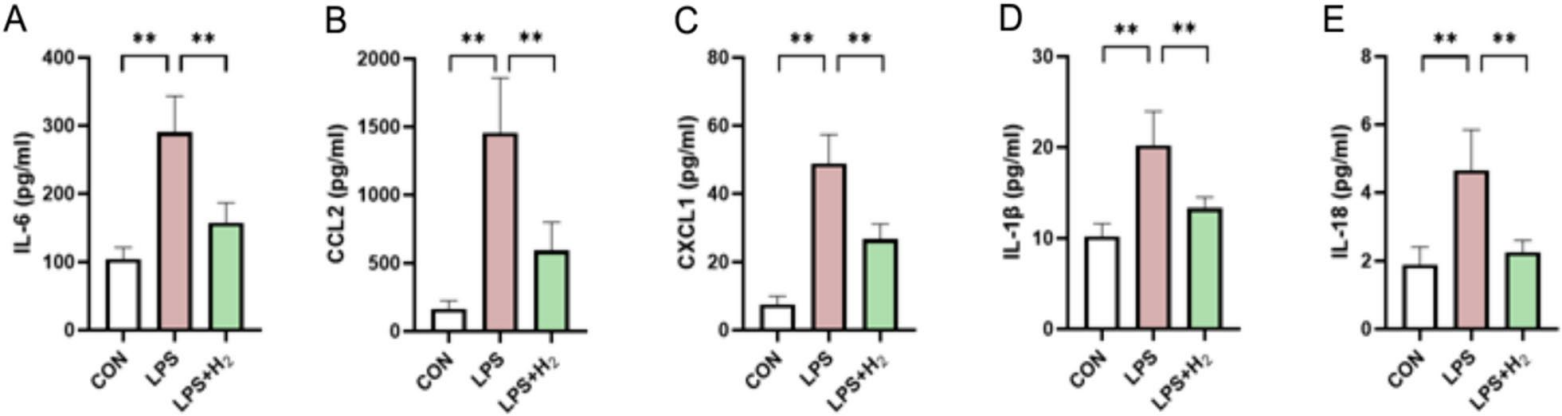
H_2_ relieved the excessive inflammatory cytokines and chemokines in LPS-induced BPD, as detected by ELISA. **A**–**E** Graphic representation of protein levels of IL-6 (**A**), CCL2 (**B**), CXCL1 (**C**), IL-1β (**D**) and IL-18 (**E**) in the placenta. Data were presented as mean ± SD of 8 samples from each group. *p < 0.05, **p < 0.01 vs LPS group

**Fig.10:**
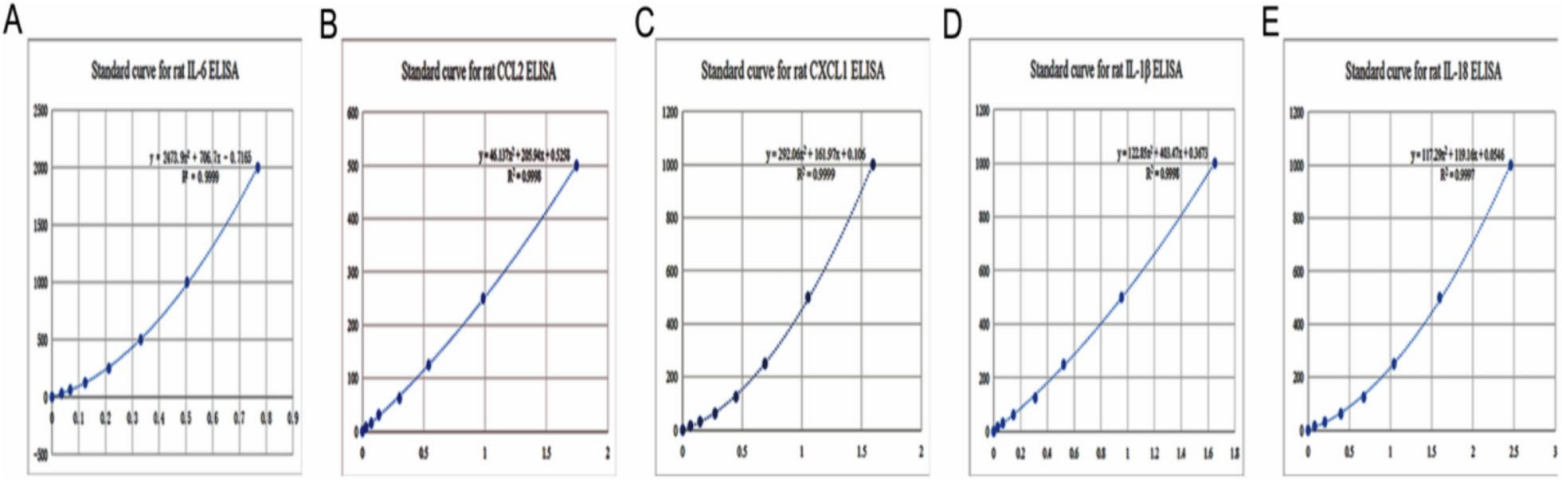
The standard curve of rat inflammatory factors by ELISA kit. A-E Represented the standard curves for IL-6 (**A**), CCL2 (**B**), CXCL1 (**C**), IL-1 β (**D**) and IL-18 (**E**), respectively

The placenta in the LPS group exhibited an inflammatory storm. For instance, inflammatory cytokines such as IL-6, IL-18, IL-1β and inflammatory chemokines such as CCL2 and CXCL1 were excessively released by placental cells (*p* < 0.05; Fig. 9A–E). When H_2_ was inhaled in the LPS group, the excessive inflammatory storm was scavenged (*p* < 0.05; Fig. 9A–E). In addition, the standard curves of rat inflammatory factors by ELISA kit are shown in Fig. 10.

### Relationship between the protein levels of TLR4–NFκB–IL6/NLRP3 signaling pathway-related proteins and inflammatory factors

**Fig. 11:**
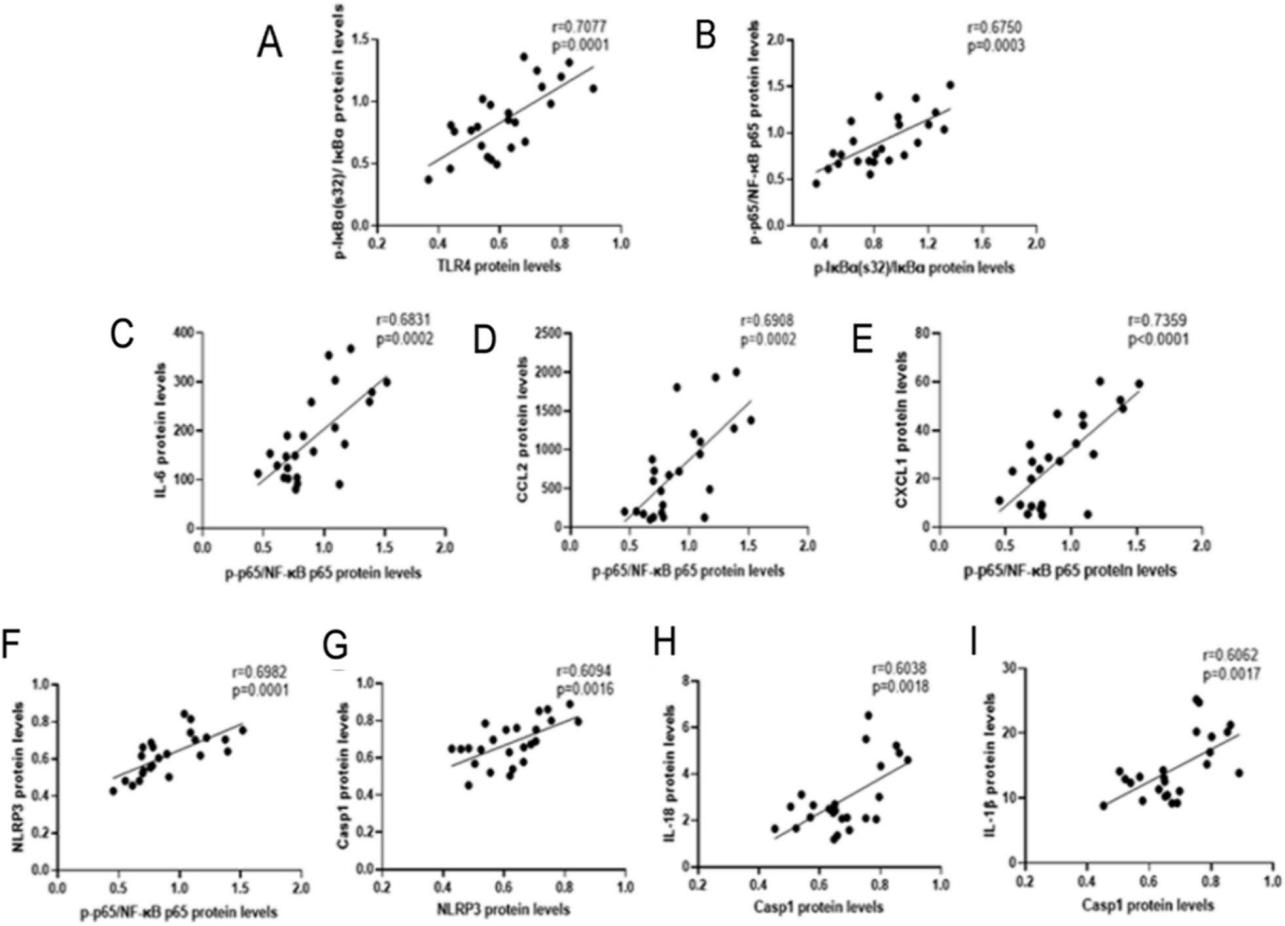
The relationships between TLR4–NFκB–IL6/NLRP3 and the protein levels of inflammatory cytokines/chemokines in placentas. **A** Relationship between TNF-α and placental p-IκBα(s32)/IκBα levels in all groups; **B** p-IκBα(s32)/IκBα vs p-p65/NF-κB p65; **C** p-p65/NF-κB p65 vs IL-6; **D** p-p65/NF-κB p65 vs CCL2; **E** p-p65/NF-κB p65 vs CXCL1; **F** p-p65/NF-κB p65 vs NLRP3; **G** NLRP3 vs Casp1; **H**–**I** Casp1 vs IL-18 and Casp1 vs IL-1β; *n* = 24, *r* = Pearson’s correlation coefficient

To verify the pivotal role of the TLR4–NFκB–IL6/NLRP3 signaling pathway in inhibiting the inflammatory response of H_2_, the study performed Pearson correlation analysis. The results showed that TLR4 was positively related to the level of degradation rate of IκBα (p-IκBα(s32)/IκBα) (*p* < 0.05; Fig. 11A), which was positively associated with the activated rate of NF-κB p65 (p-p65/NF-κB p65) (*p* < 0.05; Fig. 11B). A similar relationships were also found between p-p65/NF-κB p65 and the downstream factors including IL-6/CXCL1/CCL2 (*p* < 0.05, Fig. 11C–E). Another pathway also showed this positive correlation (*p*-p65/NF-κB p65 vs NLPR3/Casp1/IL-18/IL-1β) (*p* < 0.05, Fig. 11F–I).

## Discussion

Owing to serious complications and poor prognosis, BPD has been widely considered among LPS-induced adverse pregnancy outcomes [3]. BPD, manifested as lung injury, disrupts alveolarization and microvascular development [18]. Intra-amniotic administration of LPS at E16.5 to induce inflammatory cascades-CAM is similar to the major clinical characteristics observed in patients with BPD. LPS is derived from *E. coli* which is the most common pathogens leading to intrauterine infections in clinic, frequently applied to construct the intrauterine infection models. Intra-amniotic injection of LPS-induced CAM is one of the classic models that simulate pathological changes in BPD. The study chose to intra-amniotic injection of LPS in SD rats at E16.5 to cause intrauterine chorioamnionitis, because the lung tissue of fetal rat is in the transitional stage from tubular to vesicular stage at E16.5, which simulate and in line with the characteristics of clinical BPD (Premature infants, gestational weeks between 26 and 36 weeks, are in the vesicular stage of lung development and are prone to BPD in clinic). Intra-amniotic injection of LPS can accurately calculate the dosage of LPS according to experimental needs. However, the model still has drawbacks such as complex experimental operations, significant trauma, and susceptibility to infection. CAM can produce excessive inflammatory factors in the fetus, which affect the maturation of the fetal lung, cause fetal lung structural remodeling, and affect the contractile function of pulmonary vessels, thus leading to the occurrence of BPD [19, 20]. Our results indicated that the LPS group exhibited a disorder of intrauterine development: intrauterine fetal death (IUFD) rates were high in the LPS group. The surviving newborns also had a lower birth weight and placental weight than those in the CON group. Moreover, the results also revealed the poor outcomes of neonatal rats induced by LPS. The pups in the LPS group presented a growth-restricted state in the postnatal days, mainly in the lower body weight and lower survival ratio. Perturbed lung development (abnormal alveolarization and a severe inflammatory response) existed in LPS-induced newborns and was obviously lagging behind. Overall, LPS-induced lung injury was used as a model for BPD. Hydrogen gas is a novel therapeutic molecule, and its therapeutic potential in anti-apoptotic, anti-oxidative and anti-inflammatory effects has been recognized by many animal studies and clinical trials, especially in lung diseases [21, 22]. However, the inflammatory scavenging ability is a widely accepted mechanism of H_2_ [7, 23]. In our research, H_2_ was incorporated into the treatment of BPD as a therapeutic method. H_2_ not only attenuated the abnormal intrauterine development by reducing the IUFD and ameliorating the loss to fetus induced by LPS, but also improved the poor outcomes of neonatal rats by normalizing the LPS-induced perturbed lung development. Our findings indicated that H_2_ inhalation was indeed a promising candidate for BPD treatment, and the mechanism was deeply elucidated in this research.

Recent studies have revealed that imbalanced proinflammatory and anti-inflammatory cytokines in the placenta play a major role in the pathophysiology of BPD, characterized by excessive inflammatory response syndrome, which acts as an exposure risk factor that contributes to the development of BPD [9, 24]. Placental compositions, consisting of cytokines, chemokines and natural antioxidants, have direct consequences on the inflammatory response via anti-inflammatory, anti-bacterial and anti-viral properties [25, 26]. It is conceivable that disturbances in placental biochemical composition and in the placental signaling pathway contribute to the pathophysiology of BPD. Our results showed an excessive inflammatory response in the LPS-induced placenta in rats, and inflammatory cells were scattered in the umbilical artery. Combined with the correlation analysis results (placental weight versus birth weight), there is no doubt that placental dysfunction plays a critical role in the development of BPD.

Previous studies have confirmed that H_2_ can exert anti-inflammatory effects in a variety of cells through multiple signaling pathways. For instance, H_2_ can reduce the expression of intercellular adhesion molecules and chemokines to reduce the infiltration of neutrophils and macrophages [27, 28]. In a study of rat burns, H_2_ also alleviated the airway inflammatory response by reducing the activation of the crucial NF-κB-mediated inflammatory signaling pathway, subsequently reducing IL-1β and IL-6 levels [29]. Currently, researchers have also found the perspectives of H_2_ for coronavirus disease-2019 (COVID-19) caused by severe acute respiratory syndrome coronavirus 2 (SARS-CoV-2). COVID-19 manifests as acute inflammatory lung injury caused by a cytokine storm, H_2_ inhalation plays a notable role in annihilating inflammatory cytokines to inhibit cytokine storms. Due to the remarkable anti-inflammatory effect of H_2_, it has been recommended in acute or chronic pulmonary disease to eventually confront the COVID-19 pandemic [30–33]. Our results were consistent with a previous study: H_2_ inhalation moderated the excessive inflammatory response of LPS-treated placentas in rats, including reducing the degree of infiltration and significantly narrowing the range of infiltration. The fetal inflammatory reaction was equally mitigated upon administration of H_2_, as reflected by the umbilical artery histology.

BPD has been generally acknowledged as a complicated pathological process involving complex and redundant molecules and signaling pathways. Inflammation response is an extremely important link in the development of BPD. The placenta is a natural immune barrier for the fetus to resist pathogenic microbial infections. It can recognize pathogenic microorganisms, generate immune responses at the maternal–fetal interface, and play a role in inflammatory regulation. Placental trophoblast cells, as the first layer of immune defense, mainly express TLR4. During intrauterine infection, the expression of TLR4 mRNA in placental tissue is significantly increased and consistent with the degree of CAM, activating downstream factors IL-6, IFN-γ, IL-4 to an inflammatory cascade, ultimately leading to fetal inflammatory response syndrome. Research has found that inhibiting the expression of TLR4 in the placenta can significantly reduce the release of downstream factors, reduce inflammatory reactions, and significantly improve adverse outcomes in newborns, which has been found in hepatitis B virus infection, *E. coli* intrauterine infection and other studies. The anti-inflammatory signaling pathway mediated by TLR4 has also attracted increasing attention in diseases caused by intrauterine infections. As an important member of toll-like receptors family, TLR4, mainly stimulated by LPS, is a major inflammatory messenger that is widely involved in inflammation response. The inflammatory cascade triggered by TLR4 drives the severity of intra-amniotic inflammation in pregnancy and impacts lung development [34, 35]. NF-κB is a downstream signaling linked by TLR4. LPS could directly induce TLR4 and activation of NF-κB signaling, which in turn led to production of inflammatory factors [36, 37]. Given the role of TLR4/NF-κB signaling pathway in inflammation, we investigated the beneficial effect of inhaled H_2_ on LPS-induced BPD, and we raised the question: does hydrogen inhibit inflammation by downregulating the expression of the hub protein TLR4 and subsequently reducing the activity of the NF-κB-mediated inflammatory signaling pathway? TLR4 is stably expressed in both the first and third trimester placental trophoblast cells, recognizing LPS to initiate the intracellular cascade. Activation of TLR4 by LPS can promote phosphorylation of IκBα. In the physiological state, IκBα, as the strongest negative feedback factor in NF-κB activation, bound with NF-κB dimers (p50 with p65) in the cytoplasm to inhibit NF-κB activation. The activation of TLR4 can strengthen the phosphorylation of IκBα (p-IκBα) to get rid of the inactivation state of the NF-κB dimers. Subsequently, the exposure of the nuclear localization signal region (NLS) of NF-κB and induced the phosphorylation of NF-κB p65 (p-p65) pass into the nucleus, which could bind to KB sites on the promoters of regulatory genes to initiate gene transcription to mediate the inflammatory response. The p-p65 can enhance the mRNA transcription of IL-6, IL-1β and IL-18 to promote the production and release of proinflammatory factors. At the same time, the increased expression of IL-6 has ability to amplify the initial inflammatory signal, result in inflammatory cascade and aggravating cell damage. It can also regulate the expression of chemokines such as CXCL1 and CCL2, thus recruiting massive mononuclear macrophages and neutrophils, and triggering inflammatory infiltration and damage. Recent studies have found that TLR4 can also mediate the NLRP3-mediated inflammatory signaling pathway through the activation of NF-κB. The biological effects of the NLRP3 inflammasome in inflammatory diseases have received extensive attention. The activated NF-κB can stimulate the mRNA transcription of the NLPR3 inflammasome to a great extent in the nucleus, which can aggregate in response to cellular perturbations to drive inflammatory states. Activation of NLRP3 transforms the immature pro-caspase1 into the active effector protein caspase1 (Casp1). Casp1 can not only directly punch in the cell membrane to cause cell death, and release intracellular inflammatory factors after its lysis, but also promote the maturation and release of inflammatory cytokines IL-1β and IL-18, resulting in inflammatory cell death.

As we conceived, the toll-like receptor signaling pathway and inflammatory response biological process in placenta were strikingly located in RNA-seq results. Significantly differentially expressed genes between the LPS and LPS + H_2_ groups also revealed possible relevant downstream factors. Among them, the downstream signaling factors surrounding the NF-κB as the hub were prominently highlighted. Therefore, we identified and evaluated alterations in the TLR4/NF-κB-mediated signaling pathways in the placentas of the CON, LPS and LPS + H_2_ groups and the relevant intensity between them. The results demonstrated that H_2_ inhalation largely ameliorated the increased mRNA and protein levels of TLR4 induced by LPS in the placenta (*p* < 0.05). The same phenomenona were also reflected in the degree of IκBα degradation (p-IκBα(s32)/IκBα) and NF-κB p65 activation (p-p65/NF-κB p65) [38], the downstream activated factors NLRP3 and caspase1, concomitantly. Consistently, excessive inflammatory cytokines and chemokines in response to LPS were also scavenged by H_2_ inhalation, including IL-6, IL-18, IL-1β, CCL2 and CXCL1. Proteins are messengers of biological function, and identifying the relevant intensity of those proteins was conducive to reflecting the pivotal role of the TLR4/NF-κB-mediated signaling pathway in regulating the downstream inflammatory cytokines/chemokines [39]. The data confirmed that positive associations of TLR4 versus NF-κB and NF-κB versus the downstream signaling factors (NLRP3, Casp1, CXCL1, CCL2, IL-6) were consistent with the role of TLR4 in previous studies. The impacts on the placenta were consistent with the outcomes of neonatal lung of different groups in this study, subsequently. Administration of hydrogen reduced the LPS-induced BPD by significantly lessening placental inflammatory factors (IL-6, IL-18, IL-1β, CCL2, CXCL1, NLRP3 and Casp1) to fetal circulation, then further proceeded to alleviate the FIRS and reduced the infiltration of proinflammatory cells in fetal lung tissue, resulting in the increased amount of alveoli and the decreased area of pulmonary septum to ultimately normalize the disorder of neonatal BPD induced by LPS.

**Fig. 12:**
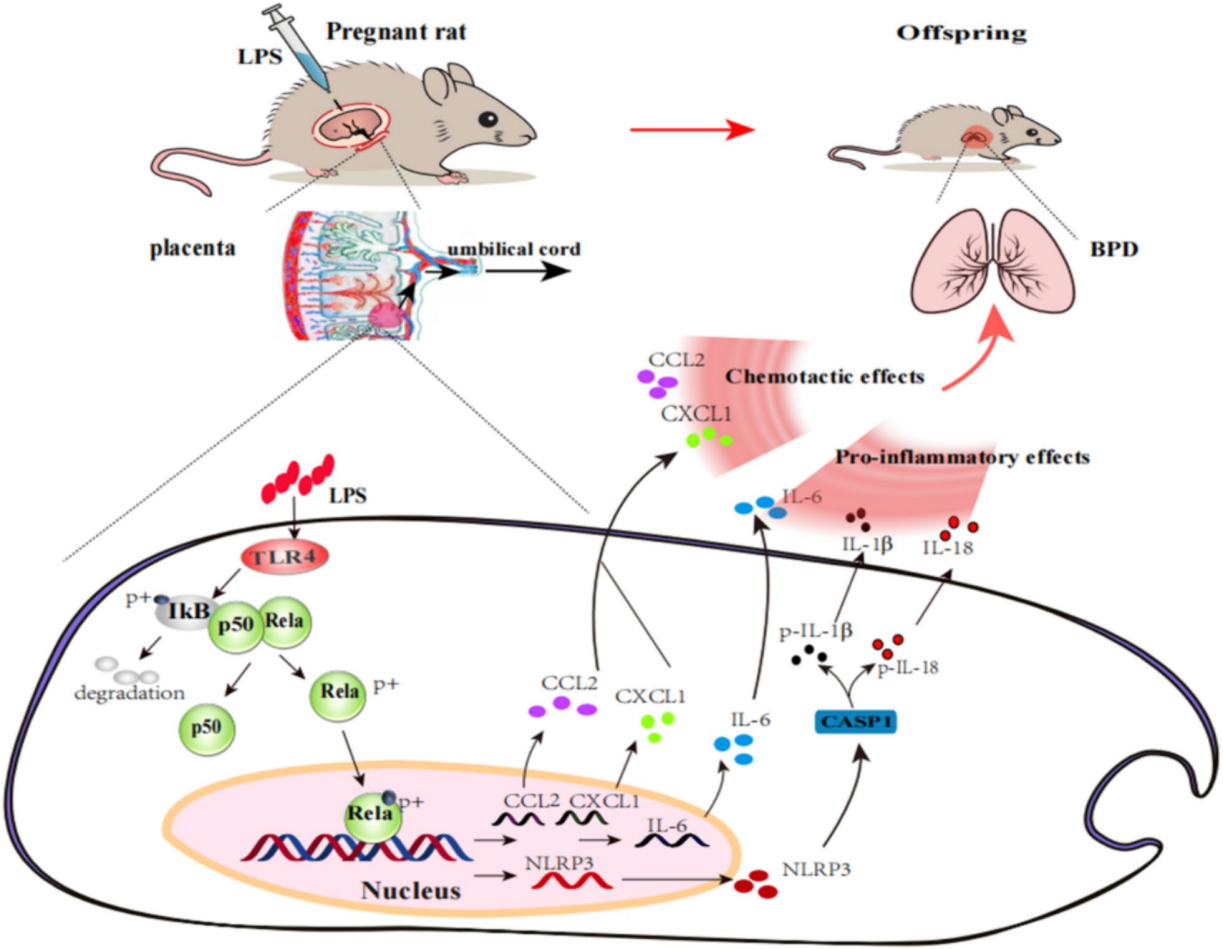
Hydrogen inhalation reduces the occurrence of LPS-induced BPD

Combined with relevant relationships, we can hypothesize that TLR4 contributes to the therapeutic effect of hydrogen inhalation on LPS-induced BPD by regulating the NF-κB-mediated inflammatory signaling pathway, resulting in the scavenging of excessive inflammatory cytokines and chemokines. The mechanism is shown in Fig. 12.

## Conclusion

In conclusion, our study demonstrated that H_2_ inhalation significantly rescued LPS-induced BPD, and the alleviation of LPS-induced BPD by H_2_ inhalation was mainly dependent on the inhibition of the TLR4–NFκB–IL6/NLRP3-mediated signaling pathway, eventually resulting in scavenging inflammatory cytokines/chemokines. Consequently, hydrogen gas inhalation under CAM in pregnancy may become an effective treatment to improve their poor outcomes.

## Data Availability

The raw RNA sequencing data supporting the conclusions of this article are available in the NCBI Sequence Read Archive (SRA) database with accession number PRJNA901070. The datasets used and analyzed during the current study are available from the corresponding author on reasonable request.

## Abbreviations

BPD: Bronchopulmonary dysplasia
H_2_: Hydrogen
LPS: Lipopolysaccharide
CAM: Chorioamnionitis
FIRS: Fetal inflammatory response syndrome
IL: Interleukin
CCL2: C–C Motif chemokine ligand 2
CXCL1: C-X-C Motif chemokine ligand 1
TLR4: Toll-like receptor 4
NF-κB: Nuclear factor kappa-B
NLRP3: NLR family pyrin domain containing 3
Casp1: Caspase1
CCL2: C–C motif chemokine ligand 2
CXCL1: C-X-C motif chemokine ligand 1
KEGG: Kyoto Encyclopedia of Genes and Genomes
TUNEL: TdT-mediated dUTP Nick-end labeling
HE: Hematoxylin–eosin
MLI: The mean linear intercept
RAC: Radial alveolar count
GO: Gene Ontology
IUFD: The intrauterine fetal death
BW: Birth weight
P14: Postnatal day 14
